# Assessment of Mitochondrial Dysfunction in Experimental Autoimmune Encephalomyelitis (EAE) Models of Multiple Sclerosis

**DOI:** 10.3390/ijms20204975

**Published:** 2019-10-09

**Authors:** Xiulin Ng, Mona Sadeghian, Simon Heales, Iain P. Hargreaves

**Affiliations:** 1 UCL Institute of Neurology, Queen Square, University College London, London WC1N 3BG, UK; 2 University Medical Center, 79106 Freiburg im Breisgau, Germany; 3 Department of Neuroinflammation, Queen Square Multiple Sclerosis Centre, UCL Institute of Neurology, London WC1N 3BG, UK; m.sadeghian@ucl.ac.uk; 4 Global Clinical Development, Actelion, High Wycombe HP12 4DP, UK; 5 Department of Molecular Neuroscience, UCL Institute of Neurology, Queen Square, London WC1N 3BG, UK; s.heales@ucl.ac.uk (S.H.); I.P.Hargreaves@ljmu.ac.uk (I.P.H.); 6 Neurometabolic Unit, National Hospital, London WC1N 3BG, UK; 7 School of Pharmacy and Biomolecular Sciences, Byrom Street, Liverpool John Moores University, Liverpool L3 3AF, UK

**Keywords:** multiple sclerosis, mitochondrial dysfunction, experimental autoimmune encephalomyelitis, fatigue and weakness

## Abstract

Multiple sclerosis (MS) is an inflammatory demyelinating disease of the central nervous system (CNS) that involves the autoreactive T-cell attack on axonal myelin sheath. Lesions or plaques formed as a result of repeated damage and repair mechanisms lead to impaired relay of electrical impulses along the nerve, manifesting as clinical symptoms of MS. Evidence from studies in experimental autoimmune encephalomyelitis (EAE) models of MS strongly suggests that mitochondrial dysfunction presents at the onset of disease and throughout the disease course. The aim of this study was to determine if mitochondrial dysfunction occurs before clinical symptoms arise, and whether this is confined to the CNS. EAE was induced in C57B/L6 mice, and citrate synthase and mitochondrial respiratory chain (MRC) complex I–IV activities were assayed at presymptomatic (3 or 10 days post first immunisation (3 or 10 DPI)) and asymptomatic (17 days post first immunisation (17 DPI) time-points in central nervous system (CNS; spinal cord) and peripheral (liver and jaw muscle) tissues. Samples from animals immunised with myelin oligodendrocyte glycoprotein (MOG) as EAE models were compared with control animals immunised with adjuvant (ADJ) only. Significant changes in MOG compared to control ADJ animals in MRC complex I activity occurred only at presymptomatic stages, with an increase in the spinal cord at 10 DPI (87.9%), an increase at 3 DPI (25.6%) and decrease at 10 DPI (22.3%) in the jaw muscle, and an increase in the liver at 10 DPI (71.5%). MRC complex II/III activity changes occurred at presymptomatic and the asymptomatic stages of the disease, with a decrease occurring in the spinal cord at 3 DPI (87.6%) and an increase at 17 DPI (36.7%), increase in the jaw muscle at 10 DPI (25.4%), and an increase at 3 DPI (75.2%) and decrease at 17 DPI (95.7%) in the liver. Citrate synthase activity was also significantly decreased at 10 DPI (27.3%) in the liver. No significant changes were observed in complex IV across all three tissues assayed. Our findings reveal evidence that mitochondrial dysfunction is present at the asymptomatic stages in the EAE model of MS, and that the changes in MRC enzyme activities are tissue-specific and are not confined to the CNS.

## 1. Introduction

Multiple sclerosis (MS) is an inflammatory demyelinating disease of the central nervous system (CNS) which has a heterogeneous clinical presentation with symptoms including impaired vision, spasms, fatigue and muscle weakness [1]. Mitochondrial dysfunction has been implicated as a contributory factor to the disease pathogenesis in MS with evidence of mitochondrial respiratory chain (MRC) dysfunction being reported in postmortem brain samples of MS patients [2] together with confocal in vivo imaging studies of the spinal cord in experimental autoimmune encephalomyelitis (EAE) mouse models of MS revealing evidence of an aberration of mitochondrial function [3]. In view of the fact that MS is widely viewed as a disorder of the CNS, its pathophysiology in tissues outside the CNS has been largely unexplored with few studies assessing systemic mitochondrial dysfunction [4]. Although clinically defined as the autoreactive T-cell attack on axonal myelin sheath, the pathophysiological mechanisms resulting in fatigue and weakness have yet to be fully elucidated [5]. Although, in view of the central role that mitochondria play in cellular energy production, there has been speculation that an impairment in the function of this organelle may contribute to the fatigue and muscle weakness presented in MS, since fatigue and weakness are hallmarks of mitochondrial dysfunction [6,7]. However, axonal loss of the corticospinal tract has been implicated as a major cause of the muscle weakness associated with MS [8]. Furthermore, structural and functional abnormalities in cortico-subcortical circuits have also been suggested as contributory factors to fatigue in MS [9], however, the exact cause of fatigue and weakness in MS still remains to be fully elucidated.

Evidence for the involvement of reactive oxygen (ROS) and reactive nitrogen (RNS) species generation [1,2,10,11,12], cytokines and other inflammatory mediators [13] in MS pathophysiology has emerged in recent years. The pathophysiology of MS includes inflammation, demyelination, remyelination, neurodegeneration and focused or diffused glial scar formation in the spinal cord or brain [14]. During the repeated inflammatory insults, mast cells and leukocytes are recruited to the site of tissue damage [15]. The subsequent respiratory burst of immune cells results in increased ROS and RNS production [16] which is thought to be a contributory factor to the mitochondrial dysfunction associated with MS, although this still requires to be fully elucidated [17,18]. However, it has been speculated that the loss of MRC function reported in this disease may arise as a result of ROS and RNS induced oxidative damage to membrane phospholipids and mitochondrial DNA [4]. The resultant MRC dysfunction may result in reduced cellular ATP production [19] that, if left uncompensated, may lead to a loss of tissue function [20,21].

The altered expression of cytokines and inflammatory mediators such as granulocyte-macrophage colony-stimulating factor (GM-CSF) [22], interferon- β (IFN-β) [23,24] and interlukin-17 (IL-17) [25] in human MS, as well as IFN-γ [26], IL-1β [27], IL-10 [28], IL-12 [29], IL-23 [30] and tumour necrosis factor-α (TNF-α) [31] in EAE models have been significantly correlated with neuroinflammation. Autoreactive T cells and B cells in the periphery can activate or differentiate into aggressive effector cells, such as Cluster of Differentiation 4+ (CD4+) T helper 1 and T helper 17 (TH1 and TH17) and CD8+ T cells, which infiltrate the blood–brain barrier (BBB), producing cytokines and inflammatory mediators which may induce repeated inflammatory insults causing gliosis, axonal demyelination and neurodegeneration [32,33].

A previous study using first day symptomatic EAE mice revealed that MRC complex I (NADH: ubiquinone reductase; EC 1.6.5.3) activity was significantly decreased at the onset of the symptomatic period and suggested that the presentation of mitochondrial dysfunction may occur before symptoms arise [3]. Therefore, in this study, we explored the possibility that MRC dysfunction may occur during the presymptomatic and asymptomatic stages of EAE. Furthermore, we also investigated whether evidence of MRC dysfunction may also be present in peripheral tissues in addition to that of the CNS.

## 2. Results

### 2.1. Spinal Cord

**MRC complex I and II/III activities were significantly altered at both presymptomatic and asymptomatic stages of disease in neurological tissue.**

**Figure 1:**
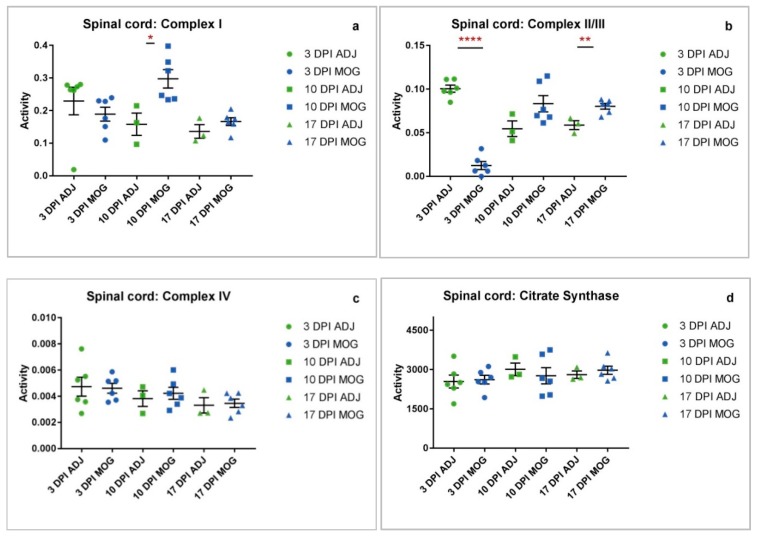
Spinal cord complex activity adjuvant (ADJ) vs. myelin oligodendrocyte glycoprotein (MOG) at different time-points. Individual raw data points for controls (ADJ) (green) and diseased (MOG) (red) have been plotted with mean and standard error of mean (SEM) to show the distribution of complex activity. Activities shown are expressed as a ratio to citrate synthase (CS) activity. (**a**) Graph showing distribution of complex I activity across ADJ and MOG. Complex I activity in MOG animals was significantly increased at 10 DPI (* *p* = 0.0209). (**b**) Complex II/III activity in MOG animals was severely decreased at 3 DPI (**** *p* < 0.0001), and significantly increased at 17 DPI (** *p* = 0.0072). (**c**) Complex IV activity showed no significant differences between MOG and ADJ animals across all 3 time-points. (**d**) Mitochondrial enrichment was measured by citrate synthase activity, which showed no significant differences in the spinal cord between MOG and ADJ animals.

Given the existing evidence for mitochondrial complex I dysfunction in symptomatic disease in neurological tissue [3], we sought to determine MRC activities at presymptomatic stages and at asymptomatic stages of the disease. Spinal cord samples from presymptomatic animals showed significantly increased complex I activity at 10 DPI of 87.9% (* *p* = 0.0209) (Figure 1a) with significantly reduced complex II/III activity at 3 DPI of 87.6% (**** *p* < 0.0001), and in asymptomatic animals significantly increased complex II/III activity at 17 DPI of 36.7% (** *p* = 0.0072) compared to control animals immunized with adjuvant only (Figure 1b).

### 2.2. Jaw Muscle

**Mitochondrial dysfunction occurs in peripheral muscle tissue at presymptomatic and asymptomatic stages of disease.**

**Figure 2:**
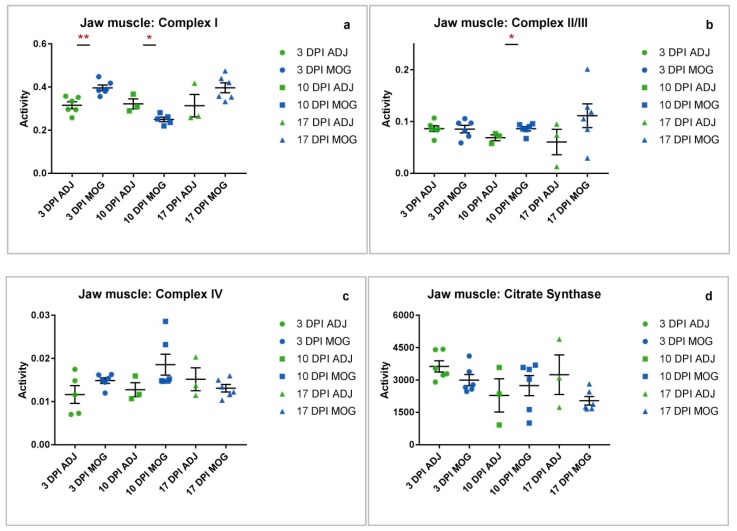
Jaw muscle complex activity ADJ vs. MOG at different time-points. Individual raw data points for controls (ADJ) (green) and diseased (MOG) (red) have been plotted with mean and standard error of mean (SEM) to show the distribution of complex activity. Activities shown are expressed as a ratio to CS activity. (**a**) Graph shows the distribution of complex I activity across ADJ and MOG. Complex I activity in MOG animals was significantly increased at 3 DPI (** *p* = 0.0029) and decreased at 10 DPI (* *p* = 0.0174). (**b**) Complex II/III activity in MOG animals was significantly increased at 10 DPI (* *p* = 0.0474). (**c**) Complex IV activity showed no significant differences between MOG and ADJ animals across all three time-points. (**d**) Mitochondrial enrichment was assessed by CS activity, which showed no significant differences in the jaw muscle between MOG and ADJ animals.

The muscle groups normally targeted in EAE according to the 7-point grading scale, adapted from Miller et al. [34] (Supplementary Figure S1), include the tail, hind limbs and fore limbs, respectively, with no apparent clinical manifestation being evident in other peripheral muscles. In view of this, we wanted to investigate the possibility that a perturbation in mitochondrial metabolism may also be occurring in muscle tissue not normally associated with EAE and asymptomatic at the time of sampling. Analysis of jaw muscle samples from presymptomatic animals showed significantly increased complex I activity at 3 DPI of 25.6% (** *p* = 0.0029), decreased complex I at 10 DPI of 22.3% (* *p* = 0.0174) (Figure 2a), and significantly increased complex II/III activity at 10 DPI of 25.4% (* *p* = 0.0474) compared to control animals immunized with adjuvant only (Figure 2b).

### 2.3. Liver

**Mitochondrial dysfunction occurs in peripheral organ tissue at presymptomatic and asymptomatic stages of disease.**

**Figure 3:**
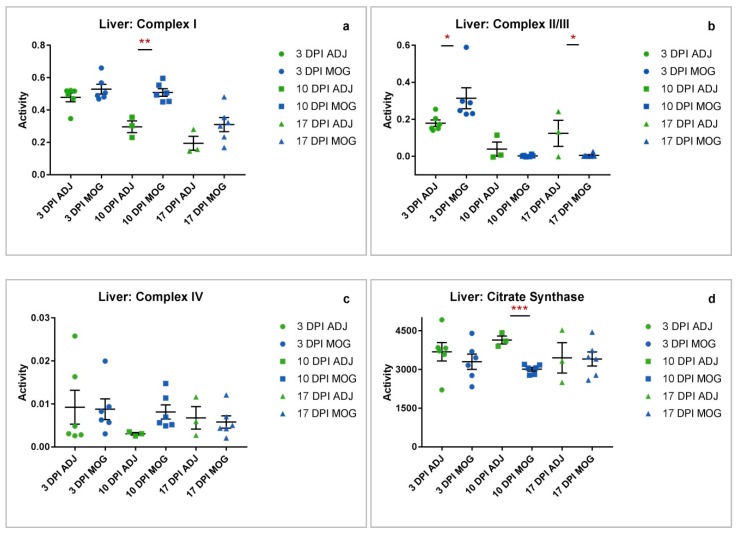
Liver complex activity ADJ vs. MOG at different time-points. Individual raw data points for controls (ADJ) (green) and diseased (MOG) (red) have been plotted with mean and standard error of mean (SEM) to show the distribution of complex activity. Activities shown are expressed as a ratio to CS activity. (**a**) Graph showing distribution of complex I activity across ADJ and MOG. Complex I activity in MOG animals was significantly increased at 10 DPI (** *p* = 0.0014). (**b**) Complex II/III activity in MOG animals was significantly increased at 3 DPI (* *p* = 0.0456) and decreased at 17 DPI (* *p* = 0.0379). (**c**) Complex IV activity showed no significant differences between MOG and ADJ animals across all three time-points. (**d**) Liver mitochondrial enrichment was assessed by determination of CS activity, which showed a significant decrease in MOG animals at 10 DPI (*** *p* = 0.0001).

As mitochondrial dysfunction was found to occur at presymptomatic stages of disease in peripheral muscle tissue, the liver was then subsequently analysed due to its physiologically high energy consumption and throughput. Assay of the liver samples from presymptomatic animals showed significantly increased complex I activity at 10 DPI (** *p* = 0.0014) (Figure 3a) and II/III activities at 3 DPI (* *p* = 0.0456), while complex II/III activity was significantly decreased in asymptomatic animals at 17 DPI (* *p* = 0.0379) compared to control animals immunized with adjuvant only (Figure 3b).

**Mitochondrial enrichment is severely compromised in peripheral organ (liver) at a presymptomatic stage of disease.**

Spinal cord, liver and jaw muscle tissues showed differing mitochondrial enrichment as indicated by CS activity (Figure 1d, Figure 2d and Figure 3d). Amongst the tissues analysed, CS activity was only significantly decreased in the liver at 10 DPI (*** *p* = 0.0001, Figure 3d). The decrease in CS, a mitochondrial marker, could reflect a loss of mitochondrial tissue density or number in MOG animals at a presymptomatic stage of EAE compared to control animals immunized with adjuvant only.

## 3. Discussion

The findings of this study have revealed evidence that mitochondrial dysfunction is not limited to the CNS with evidence of MRC dysfunction also being detected in the jaw muscle and liver of the EAE mouse model of MS, therefore providing evidence that EAE and MS may be associated with systemic mitochondrial dysfunction. The majority of the compromised MRC enzyme activities detected presented at the early, presymptomatic stages of the disease in the EAE mouse indicating that MRC dysfunction may precede clinical symptoms. Statistically significant observations were found to occur mainly in MRC complex I activity and complex II/III activity across all three tissues (spinal cord, jaw muscle and liver), whereas complex IV activity showed little or no variation between the control and MOG animals throughout the disease course. A possible reason to account for the apparent preservation of MRC complex IV activity may reflect the time course of the present study with the final tissue samples being taken 17 days DPI; this may have been an insufficient period of time to detect any evidence of an impairment in enzyme activity and future studies may be required to elucidate this possibility. Furthermore, other studies have alluded to the relative stability of complex IV with a study by Osellame et al. [35] reporting evidence of decreased complex I and II/III enzyme activities whilst the activity of complex IV was found to be unaffected in brain tissue from a mouse model of Gaucher disease. Significant changes in MRC complex I activity were found to occur only at presymptomatic stages (in spinal cord increased * at 10 DPI; in jaw muscle increased ** at 3 DPI and decreased * at 10 DPI; in liver increased ** at 10 DPI), while significant changes in complex II/III activity occurred at both presymptomatic and asymptomatic stages of the disease (in spinal cord decreased **** at 3 DPI and increased ** at 17 DPI; in jaw muscle increased * at 10 DPI; in the liver increased * at 3 DPI and decreased * at 17 DPI). Assessment of CS activity, a biochemical marker of mitochondrial enrichment [36] indicated no considerable differences between the tissues except at 10 DPI in the liver where it was significantly decreased in MOG animals compared to those immunized with adjuvant (ADJ) only. The reason for this difference has yet to be elucidated.

MS is defined as a chronic inflammatory demyelinating disease of the CNS [37], and for this reason a preponderance of the research on this disease has focused on the CNS in symptomatic disease models. Inflammation caused by autoreactive T lymphocyte attack on the myelin sheath [5] leads to mast cell and leukocyte recruitment, which triggers respiratory burst and ROS and RNS generation [16]. Excessive ROS generation has been intimately linked with caspase activation and cytochrome c release resulting in apoptosis [38]. The resulting oxidative damage induced by ROS and RNS on mitochondrial membrane lipids, MRC enzyme complexes and mtDNA may also cause an impairment of mitochondrial function [39]. The resulting mitochondrial dysfunction may also result in decreased ATP production [19,40] which may underlie MS related fatigue and contribute to the muscle weakness reported in both EAE and MS [6,7]. However, the primary cause of muscle weakness in EAE and MS has been reported to be the result of the axonal loss in the corticospinal tract [8]. A significant increase in complex I activity was observed as early as 3 DPI in the jaw muscle, as well as at 10 DPI in the spinal cord and liver of the EAE mouse, possibly in an effort to compensate for the impairment of the other MRC enzyme complexes and maintain homeostasis in oxidative phosphorylation in the diseased state as has been observed in other disorders where a pattern of impaired and increased MRC enzyme activities has been reported [41,42].

Interestingly, whilst MRC complex I activity was found to be increased at 10 DPI in the spinal cord, complex II/III activity was already markedly decreased at the early time-point of 3 DPI. A possible reason for the decreased complex II/III activity may be an underlying deficit in coenzyme Q10 status which has been suggested by the ability of this isoprenoid when used as a supplement to improve fatigue and depression in MS patients [43]. The activity of MRC complex II/III is dependent upon the endogenous level of the electron carrier, coenzyme Q10 for its activity, and therefore a decrease in coenzyme Q10 status may impair complex II/III activity [44]. However, the differing MRC enzyme activities may simply reflect the levels of ROS and RNS present at these two time-points and their resultant effect on the enzyme activities [39]. This may also be an explanation for the observed increase in complex II/III activity at 17 DPI (**; 36.7%) in the spinal cord. However, it may also be a mechanism to increase oxidative phosphorylation capacity and compensate for its initial marked decrease at 3 DPI in this tissue [41,42].

Interestingly, a study by Sadeghian et al. has reported evidence of impaired MRC complex I activity in the spinal cord of EAE mice, whilst the activity of complex II/III remained unchanged [3]. In contrast, the present study has observed both a non-significant increase in complex I activity and a significant increase in complex II/III activity in the spinal cord of the EAE mice. A possible explanation for the apparent disparity in results is that in the study by Sadeghian et al. [3] spinal cord samples were taken during the symptomatic stage which was defined as between 9–14 DPI whereas in the present study, spinal cord samples were taken from asymptomatic diseased animals at 17 DPI. Although in EAE, the mechanism by which inflammation may affect the activities of MRC enzymes has yet to be fully elucidated, ROS and RNS have been implicated to be involved [4,10,11]. Therefore, the possibility arises that during the symptomatic period the levels of RNS and ROS may be raised and capable of impairing MRC enzyme activity [3,4,39]. Consequently, during the asymptomatic period the level of ROS and RNS may fall allowing recovery of MRC enzyme activities [3,4,39].

In addition to causing an impairment of MRC enzyme activities [1], RNS have also been reported to cause increased MRC complex I and III activities by as of yet unknown mechanisms [45], although it has been suggested that this may result from an induction in gene transcription since RNS are able to act as intracellular signaling molecules [42]. Therefore, the level of RNS present at 17 DPI may have been sufficient to induce an increase in the transcription of MRC complex I and complex II/III resulting in the increased activities of these enzymes observed at this time-point in the present study, although this requires a further study to confirm or refute this mechanism.

As previously mentioned, the determination of MRC activity in the jaw muscle of the EAE mouse revealed an increased MRC complex I activity at 3 DPI (**; 25.6%). At 10 DPI, the activity of complex I was found to decrease (*; 22.3%) in the jaw muscle paralleling an increase in complex II/III activity at this time-point (*; 25.4%). The latter increase in complex II/III activity is possibly required to offset the decrease in complex I activity [41,42]. The indicated loss of MRC complex I activity at 10 DPI in jaw muscle could be attributed to an accumulation of ROS and RNS at this time-point sufficient to impair the activity of this enzyme [3,4,46]. Studies have yet to be undertaken to confirm this suggested mechanism, however, there is strong evidence to suggest that the neutralization of ROS and RNS can rescue nitrated MRC complex I restoring ATP production to normal levels [47].

In contrast to the jaw muscle, whose MRC complex I activity was found to decrease at 10 DPI, liver complex I activity was found to be significantly increased at the same time-point, suggesting that the changes in MRC activities may be tissue-specific. This increase could serve as a compensatory mechanism triggered by reduced tissue oxygen perfusion or insufficient ATP production in the tissue [47,48,49]. This may also be an explanation for the increased liver MRC complex II/III activity detected at 3 DPI (*; 75.2%), or again, it may reflect the level of RNS exposure of the tissue [41,42,43]. At subsequent time-points, liver MRC complex II/III activity was found to decrease, being significantly reduced (*; 95.7%) at 17 DPI compared to the control animals. The cause of the loss of MRC complex II/III activity in liver at these later time-points is uncertain, however, it may be as a result of exposure to RNS and ROS which has been reported in EAE [3] and which may cause a loss of MRC enzyme activity as a possible consequence of oxidative damage to mitochondrial membrane lipids, protein subunits of the enzyme complexes or mitochondrial DNA [4,39].

An important observation from the present study is that the MRC enzyme activities differ between spinal cord, liver and jaw muscle at the respective time periods. This may suggest differing sensitivities of the tissue to the inflammatory response in EAE and possibly reflect differences in antioxidant status between the tissues. Another notable observation from the present study is that MRC complex IV activity in spinal cord, jaw muscle and liver was found to be comparable between the MOG and ADJ controls. Although this is in contrast to other studies where impairment of complex IV activity has been reported in EAE [1] and human tissue from MS patients [50], numerous factors such as the period of the disease course, postmortem delay and tissue type may have a bearing on the results of the enzyme activity assessments [19,51]. However, the consistency of complex IV activity data (Figure 1c, Figure 2c and Figure 3c) for both ADJ and MOG in all tissues assayed in this study supports the reliability of the results obtained for this investigation.

The findings of this study have indicated evidence of tissue- and stage-specific changes in the MRC enzyme activities of the EAE mouse model of MS. In view of the possibility that the energy thresholds may vary between tissues as indicated by the study by Davey et al. (1998) [40], that is, the level to which the MRC enzymes must be inhibited before oxidative phosphorylation is impaired, the results of the present study indicate that energy levels may vary between different tissues during the course of the disease. However, the effect this may have on cellular ATP synthesis requires further investigation in the EAE mouse model and will form the basis of a further study. Therefore, although we indicated evidence of MRC dysfunction in the present study, the level of mitochondrial impairment may not be sufficient to result in organ dysfunction at the stage of the disease investigated. Further work will be required to elucidate the clinical consequences of the mitochondrial dysfunction identified in the asymptomatic stages of EAE.

The increase in tissue MRC enzyme activities determined during the pre- and asymptomatic stages of disease in the EAE mice as well as potentially being a mechanism to compensate for impaired MRC function [42] may also result in an increase generation of ROS species which may contribute to the impaired MRC function [39] as reported in the later symptomatic stages of the disease [3]. Furthermore, these tissue specific changes in MRC enzyme activities in both the CNS and peripheral tissues may also be contributory factors to tissue specific pathologies occurring in the EAE mouse as well as in MS patients [51], although this has yet to be investigated. A previous study by Hargreaves et al. [4] detected evidence of MRC complex IV deficiency in blood mononuclear cells isolated from MS patients, further supporting the possibility that non-CNS mitochondrial dysfunction may also be present in this disease. Furthermore, evidence of liver dysfunction has been reported in a rat model of EAE suggesting that clinical symptoms may not be confined to the CNS in this condition [52].

The loss of MRC enzyme activity reported in the present study is possibly caused by the release of pro-inflammatory cytokines and not as the result of a primary genetic disorder. Pro-inflammatory cytokines have been reported to cause the upregulation of inducible nitric oxide synthase (iNOS) activity with a concomitant increase in RNS generation which can result in an impairment of MRC enzyme activity [53]. This is further supported by the study of Hargreaves et al. which found that MS patients treated with beta interferon, a known nitric oxide synthase inhibitor had higher levels of MRC complex IV activity than untreated patients [4].

Overall, the findings of this study in conjunction with those of the studies by Lu et al. [2], Van Horssen et al. [10] and Sadeghian et al. [3] have indicated evidence of MRC dysfunction in EAE and MS, although this impairment in MRC function may not be confined to the CNS. Importantly, this study for the first time has shown evidence of MRC impairment during the presymptomatic stages of EAE, preceding clinical symptoms, and therefore indicates the need to consider therapeutic strategies that target mitochondrial dysfunction during the early stages of the disease.

## 4. Materials and Methods

### 4.1. Animals

C57B/L6 female mice weighing 20 g each were purchased from Harlan, UK, and were housed and bred in pathogen-free animal holding rooms in compliance with the UK Home Office Animals (Scientific Procedures) Act (1986) and approved by the local ethics committee (University College London, UK, January 2016). EAE was induced in the disease group (“MOG”) mice at 10–16 (mean = 14) weeks old, while the control group (“ADJ”) mice were immunized using adjuvant only.

### 4.2. EAE Induction

In total, 30 mice were used in this study. 12 adjuvant (ADJ) control and 18 myelin oligodendrocyte glycoprotein (MOG)-induced female C57B/L6 mice weighing 20 g each were induced at 10–16 (mean = 14) weeks of age. Immunisation protocol was as follows. Day 0: Mice were anaesthetized with 2% isofluorane. ADJ control mice received two doses (100 µL per dose) of an emulsion containing 200 µl of Incomplete Freund’s adjuvant (IFA) (Sigma, Gillingham, Dorset, UK) supplemented with 5 mg/mL heat-killed mycobacterium (MTB) made up in 1:1 ratio with saline by subcutaneous injection, followed by an intraperitoneal injection of pertussis toxin (50 ng/mL) (Calbiochem, Nottingham, UK). MOG mice received two doses (100 µL per dose) of an emulsion containing 200µg of MOG 35-55 (2 mg/mL stock concentration) made up in 1:1 ratio with Incomplete Freund’s adjuvant (IFA) (Sigma, Gillingham, Dorset, UK) supplemented with 5 mg/mL heat-killed mycobacterium (MTB), followed by an intraperitoneal injection of pertussis toxin (50 ng/mL) (Calbiochem, Nottingham, UK). Day 2: The pertussis injection was repeated intraperitoneally. Day 7: Mice were re-immunised with MOG 33-35/CFA emulsion administered subcutaneously.

Mice were weighed daily and scored on clinical signs of neurological deficit using a 7-point grading scale, adapted from Miller et al. [34] (Supplementary Figure S1). Presymptomatic mice for the 3 DPI and 10 DPI time-points, being without neurological symptoms, were not given a score on the 7-point grading scale as the scale scores are based on signs of neurological deficit. Signs of neurological deficit appeared from 12 DPI, reaching hind-limb paralysis (score 4 of the 7-point grading scale (Supplementary Figure S1) at around day 18. Mice at 17 DPI are therefore expected to be symptomatic (score 4), but only neurologically asymptomatic EAE-induced mice were selected for this time-point in this study. Asymptomatic mice selected for the 17 DPI time point, being without neurological symptoms, were therefore also not given a score on the 7-point grading scale for neurological deficit.

### 4.3. Sample Collection

Tissue samples were collected at 3 time-points: 3 days post first immunisation (3 DPI), 10 days post first immunisation (10 DPI), and 17 days post first immunisation (17 DPI). Spinal cord, liver and jaw muscle were placed in Eppendorf tubes and frozen immediately in liquid nitrogen (N2), and transferred for storage at −80 °C.

### 4.4. Storage and Transport

Samples were sorted and transported on dry ice. On arrival at the destination laboratory, samples were frozen immediately at −80 °C.

### 4.5. Tissue Homogenization

The tissue sample was obtained from the animal, frozen at the table, stored at −80 °C and couriered to the destination laboratory on ‘dry ice’. The muscle biopsy was homogenized on ice, using a pre-chilled glass hand-held homogenizer. Briefly, the tissue sample (50–100 mg) was homogenized 1:9 (*w*/*v*) in: 320 mmol/L, 1 mmol/L ethylenediamine tetra acetic acid dipotassium salt, and 10 mmol/L Trizma-base, pH 7.4 [45].

### 4.6. Enzymatic Determinations

Enzymatic determinations were undertaken at 30 °C using a Uvikon XL spectrophotometer (Northstar, Leeds, UK).

The specific activities of the MRC enzymes were determined according to previously described methods:

Complex I activity was measured by the rotenone sensitive reduction of NADH at 340 nm as described by the method of Ragan et al. [54]. Rotenone is a specific inhibitor of complex I activity, and therefore in the assay, the enzymatic rate pre rotenone addition is subtracted from the activity post rotenone addition to provide a complex I specific enzyme activity [54].

Complex II/III (succinate dehydrogenase: cytochrome *c* reductase; EC. 1.3.5.1 + 1.10.2.2) was measured by the antimycin A sensitive reduction of cytochrome c at 550nm according to the method of King [44]. Antimycin A is a specific inhibitor of complex III activity and therefore in the assay, the enzymatic rate pre antimycin A addition is subtracted from the activity post antimycin A addition to provide a complex I–III specific enzyme activity [44].

Complex IV (cytochrome c oxidase; EC 1.9.3.1) activity was measured by the potassium cyanide (KCN) sensitive oxidation of reduced cytochrome c at 550 nm according to the method of Wharton and Tzagoloff [55]. KCN is a specific inhibitor of complex IV and therefore the assay was run twice, once with KCN and once without KCN, the enzymatic rates of the two assays were subtracted to provide a complex IV specific enzyme activity [45].

Citrate synthase (CS; EC 2.3.3.1) activity was determined by the method of Shepherd and Garland [56]. MRC enzyme activities were expressed as a ratio to CS to correct for mitochondrial enrichment of the samples [57].

### 4.7. Lowry Total Protein Assay

Protein was determined according to the method of Lowry and colleagues [58] using bovine serum albumin as a standard.

### 4.8. Stastical Analysis

Citrate synthase, complex I, complex II/III and complex IV activities were calculated from the absorbances recorded in the assays and analysed with Microsoft Excel (Microsoft, Redmond, WA, USA) and GraphPad Prism (GraphPad, San Diego, CA, USA). Unpaired *t*-tests were carried out for each set of data, and *p* < 0.05 was considered significant (* *p* < 0.05; ** *p* < 0.01; *** *p* < 0.001). Comparison was made between ADJ and MOG animal groups.

## Supplementary Materials

Supplementary materials can be found at https://www.mdpi.com/1422-0067/20/20/4975/s1.

## Abbreviations

ADJ	Adjuvant
ATP	Adenosine Triphosphate
BBB	Blood–Brain Barrier
CD	Cluster of Differentiation
CFA	Complete Freund’s Adjuvant
CNS	Central Nervous System
CS	Citrate Synthase
DPI	Days Post First Immunisation
EAE	Experimental Autoimmune Encephalomyelitis
GM-CSF	Granulocyte-Macrophage Colony-Stimulating Factor
IFA	Incomplete Freund’s Adjuvant
IFN	Interferon
IL	Interlukin
KCN	Potassium Cyanide
MOG	Myelin Oligodendrocyte Glycoprotein
MRC	Mitochondrial Respiratory Chain
MS	Multiple Sclerosis
MTB	Mycobacterium Tuberculosis
mtDNA	Mitochondrial DNA
NADH	(Reduced) Nicotinamide Adenine Dinucleotide
NADPH	(Reduced) Nicotinamide Adenine Dinucleotide Phosphate
nDNA	Nuclear DNA
RNS	Reactive Nitrogen Species
RNSROS	Reactive Oxygen Species
TCA	Tricarboxylic acid cycle
TH	T-helper
TNF-α	Tumour Necrosis Factor-alpha
