# Supplementary material for: Assessment of Mitochondrial Dysfunction in Experimental Autoimmune Encephalomyelitis (EAE) Models of Multiple Sclerosis
Source: Int J Mol Sci. 2019 Oct 9;20(20):4975. doi: 10.3390/ijms20204975 (PMC6829485; doi:10.3390/ijms20204975)
Supplement: Supplementary file 1 [file ijms-20-04975-s001.pdf]

Clinical Sign	Score
• Slightly flaccid tail<br>• Slight tail spasticity	1
• Flaccid tail<br>• Tail spasticity	1.5
• Slightly impaired righting reflex	2
• Impaired righting reflex	2.5
• Slight hind limb weakness<br>• Unilateral hind limb weakness<br>• Slight hind limb spasticity<br>• Unilateral hind limb spasticity	3
• Bilateral hind limb weakness<br>• Bilateral hind limb spasticity	3.5
• Weakness in one hind limb, paralysis in the other<br>• Severe bilateral hind limb weakness<br>• Hind limb spasticity causing immobility in same limb<br>• Severe bilateral hind limb spasticity	4
• Hind limb paralysis<br>• Hind limb spasticity with both limbs paralysed	4
• Slight fore limb spasticity<br>• Unilateral fore limb weakness	4.5
• Fore limb weakness<br>• Fore limb spasticity	5
• Weakness in one fore limb, paralysis in the other<br>• Severe fore limb weakness<br>• Spasticity in one fore limb, paralysis in the other<br>• Severe fore limb spasticity	5.5
• Bilateral fore limb paralysis<br>• Bilateral fore limb spasticity and immobility	6.5
• Moribund or dead	7

Supplementary Figure S1. Clinical scoring table for signs of neurological deficits in murine models, adapted from Miller et al. [56]. Clinical signs for neurological deficits in mice are matched with a score for the determination of severity of disease. In this study, mice were scored on experimental autoimmune encephalomyelitis (EAE) clinical symptoms. Presymptomatic mice for the 3 DPI and 10 DPI time-points were ensured free of neurological deficits. Signs of neurological deficit appeared from 12 DPI, reaching hind-limb paralysis (score 4 of the 7-point grading scale) at around day 18. Mice at 17 DPI are therefore expected to be symptomatic (score 4), but only neurologically asymptomatic mice were selected for this time-point in this study, demonstrating that although no outward symptoms present in these mice induced with EAE, metabolic functions are altered.
